# EXPLORING RELATIONSHIPS BETWEEN RELIGIOUS COPING AND GENERAL COPING STRATEGIES IN OLDER LONG-TERM CARE RESIDENTS

**DOI:** 10.1093/geroni/igz038.1861

**Published:** 2019-11-08

**Authors:** Nathaniel Andrew, Suzanne Meeks

**Affiliations:** University of Louisville, Louisville, Kentucky, United States

## Abstract

Aging long-term care residents utilize a variety of coping strategies to manage stressors. While many older adults report religious faith is important in the coping process, it is unclear how religious coping appraisals and religious coping styles fit within a broader coping framework. This poster explores relationships between religious coping and general coping strategies in a convenience sample of older nursing home and assisted living residents (median age = 71.5). In this cross-sectional study, we interviewed residents (N = 102) from long-term care facilities (N = 11) in the Louisville metropolitan area and southern Indiana. Participants responded to questions about religious practices, religious coping, general coping, stress, life satisfaction, psychological distress, and health. The present analyses examined correlations between religious coping appraisals/styles and general coping strategies. We found: 1) small to moderate associations between theoretically adaptive religious coping appraisals/styles (e.g., positive appraisals, collaborative/deferring styles) and theoretically adaptive general coping strategies (e.g., positive reframing, instrumental support), and 2) small to moderate associations between theoretically maladaptive religious coping appraisals/styles (e.g., negative appraisals, self-directing styles) and theoretically maladaptive general coping strategies (e.g., denial, behavioral disengagement). Our results identify interesting conceptual relationships suggesting residents who report positive religious coping appraisals and less independent religious coping styles use adaptive coping strategies more frequently. These constructs may be explored in future research through examining their theoretical uniqueness and whether they independently account for variance in clinically-relevant outcomes. Further study of religious coping in these settings may help promote resilience and optimal aging for long-term care residents.

